# Rabbit VX2 Liver Tumor Model: A Review of Clinical, Biology, Histology, and Tumor Microenvironment Characteristics

**DOI:** 10.3389/fonc.2022.871829

**Published:** 2022-05-10

**Authors:** Florentina Pascale, Jean-Pierre Pelage, Michel Wassef, Saïda H. Ghegediban, Jean-Pierre Saint-Maurice, Thierry De Baere, Alban Denys, Rafael Duran, Frédéric Deschamps, Olivier Pellerin, Noboru Maeda, Alexandre Laurent, Julien Namur

**Affiliations:** ^1^Research and Development Department, Archimmed Société à responsabilité limtée Limited liability Company (SARL), Jouy-en-Josas, France; ^2^Université de Caen Normandie (UNICEAN), Centre d'Energie atomique (CEA), Centre National de la Recherche Scientifique, Imagerie et Stratégies Thérapeutiques pour les Cancers et Tissus Cérébraux CERVOxy (ISTCT-CERVOxy) Normandie University, Caen, France; ^3^Department of Interventional and Diagnostic Imaging, University Hospital of Caen, Avenue de la Côte de Nacre, Caen, France; ^4^Service d’Anatomie et Cytologie Pathologiques, Hôpital Lariboisière, Assistance Publique Hopitaux de Paris (APHP); Unité de Formation et de Recherche (URF) de Médecine Paris Nord, Université de Paris, Paris, France; ^5^Department of Neuroradiology, Hôpital Lariboisière, Assistance Publique Hopitaux de Paris (APHP); Unité de Formation et de Recherche (URF) de Médecine Paris Nord, Université de Paris, Paris, France; ^6^Department of Interventional Radiology, Gustave Roussy Cancer Center, Villejuif, France; ^7^Unité de Formation et de Recherche (URF) Médecine Le Kremlin-Bicêtre, Université Paris-Saclay, Le Kremlin-Bicêtre, France; ^8^Department of Radiology and Interventional Radiology, Centre Hospitalier Universitaire Vaudois, University of Lausanne, Lausanne, Switzerland; ^9^Department of Interventional Radiology, Hôpital Européen Georges Pompidou, Assistance Publique Hopitaux de Paris (APHP) Université de Paris, Paris, France; ^10^Department of Diagnostic and Interventional Radiology, Osaka International Cancer Institute, Osaka, Japan

**Keywords:** embolization, locoregional treatments, imaging, immune oncology, angiography, tumor microenvironment

## Abstract

The rabbit VX2 is a large animal model of cancer used for decades by interventional radiologists to demonstrate the efficacy of various locoregional treatments against liver tumors. What do we know about this tumor in the new era of targeted therapy and immune-oncology? The present paper describes the current knowledge on the clinics, biology, histopathology, and tumor microenvironment of VX2 based on a literature review of 741 publications in the liver and in other organs. It reveals the resemblance with human cancer (anatomy, vascularity, angiogenic profile, drug sensitivity, immune microenvironment), the differences (etiology, growth rate, histology), and the questions still poorly explored (serum and tissue biomarkers, genomic alterations, immune checkpoint inhibitors efficacy).

## Introduction

The rabbit VX2 is a well-known animal tumor model in interventional radiology. Initially developed by Kid and Rous in the late 1930s (1), it is an anaplastic squamous cell carcinoma derived from *Shope papillomavirus* infection in rabbit. The tumor can be serially transplanted from one animal to another by allograft implantation and may grow in any organ or grafted tissue. A distinctive feature of VX2 is the fact that it does not request genetically modified or immunocompromised subjects but can be transplanted to normal immunocompetent animals. Sizeable tumors are obtained within a couple of weeks with up to >95% efficiency and good reproducibility. Another reason why interventionalists have been using the rabbit VX2 is the larger size of the animal compared to the conventional rodent tumor model allowing the use of similar medical devices and an interventional procedure as in patients. For decades, VX2 has been used successfully to demonstrate the efficacy of various locoregional treatments such as transarterial chemoembolization (TACE), thermoablative therapies, radioembolization, and combinatory approaches (2–7).

The first immune checkpoint inhibitors have just entered treatment algorithms for primary and metastatic liver tumors (8), and many trials combining immunotherapies and locoregional treatments have been initiated (9). In this new era, researchers expect more from their translational model besides a growing mass that responds to physical or chemical aggression. Is the VX2 tumor a relevant model to evaluate these new tools and to what extent? The answer is not straightforward.

Among the 1,487 publications related to the VX2 model in the PubMed database, basic research articles are very few. Most investigative papers focus on the safety or efficacy of a new treatment and give a sparse and superficial description of the tumor. However, this literature search also reveals many trials, past and recent, that used the model and provides significant information about tumor growth characteristics, its biology, its genetic expression, or even its immunobiology.

The present paper will give a comprehensive review of the current knowledge on the VX2 tumor model with regard to clinical and imaging characteristics, macro- and microvascularization, histopathology, and microenvironment.

## 1 Clinics

### 1.1 Tumor Induction, Growth, and Spread

The methods to induce the development of VX2 tumors in the liver have been described in detail in several review and investigation papers (10–12). Different types of inoculum and modalities of grafting of the tumor cells into the liver may be used that will affect tumor growth and tumor seeding. Briefly, the implantation of fragments dissected from fresh tumor grown in the muscle of a donor animal and placed surgically in the liver by laparotomy gives a satisfactory tumor take rate (90%–100%) and limits the risk of early extrahepatic dissemination. It is most commonly used in trials evaluating locoregional therapies. On the other hand, the injection of a cell suspension seems to provide a lower tumor take rate, accelerated tumor development, and earlier development of lung metastases due to accidental infusion of tumor cells into vessels. This approach may be considered for studying the antitumor effects of systemic therapies.

**Figure 1:**
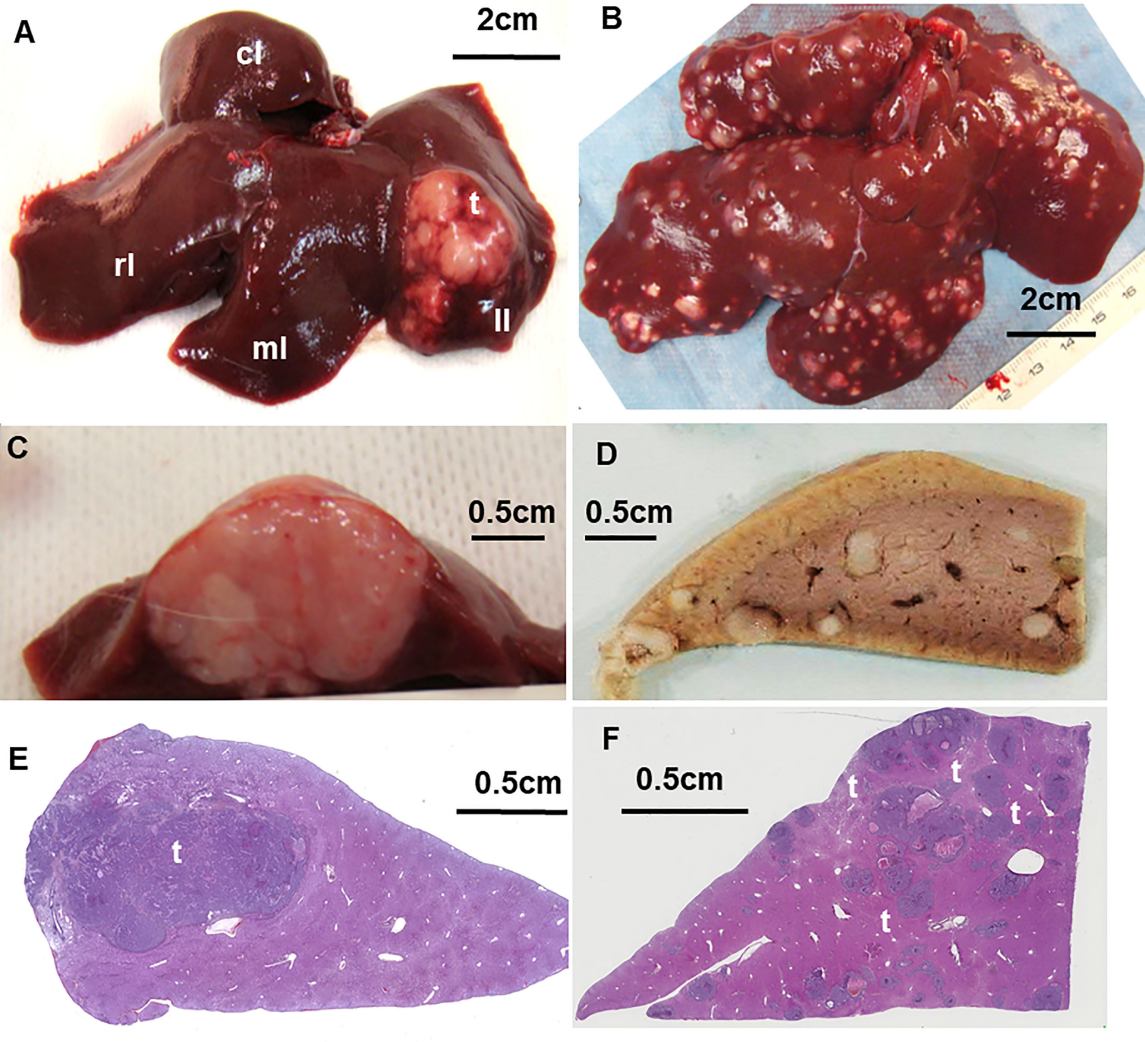
**(A)** Macroscopic aspect of the uninodular VX2 tumor into the left lobe of the liver and **(B)** multinodular tumors, explanted at D14 after grafting. **(C)** Cross section of the uninodular tumor into the left liver and **(D)** of the multinodular liver lobe fixed in formalin. **(E)** Digitized histology section of the liver left lobe bearing uninodular or multinodular tumors **(F)** of different sizes stained with hematein–eosin–saffron showing the tumor in liver parenchyma. T, tumor; LL, left lobe; ML, median lobe; RL, right lobe; CL, caudate lobe. From (16, 17).

Regarding the number of tumor nodules, the large majority of studies induce a single VX2 tumor because it is easier to create and follow. Multiple VX2 nodules can still be grown successfully, either by implanting several fragments in different locations of the organ or by injecting the inoculum in a vessel irrigating the liver (13–16) (**Figures 1A–F**). These approaches have been developed to mimic metastases to the liver. They also seem relevant as a model of primary cancer since the population treated by locoregional or systemic therapies are generally patients with multifocal disease (18).

**Figure 2:**
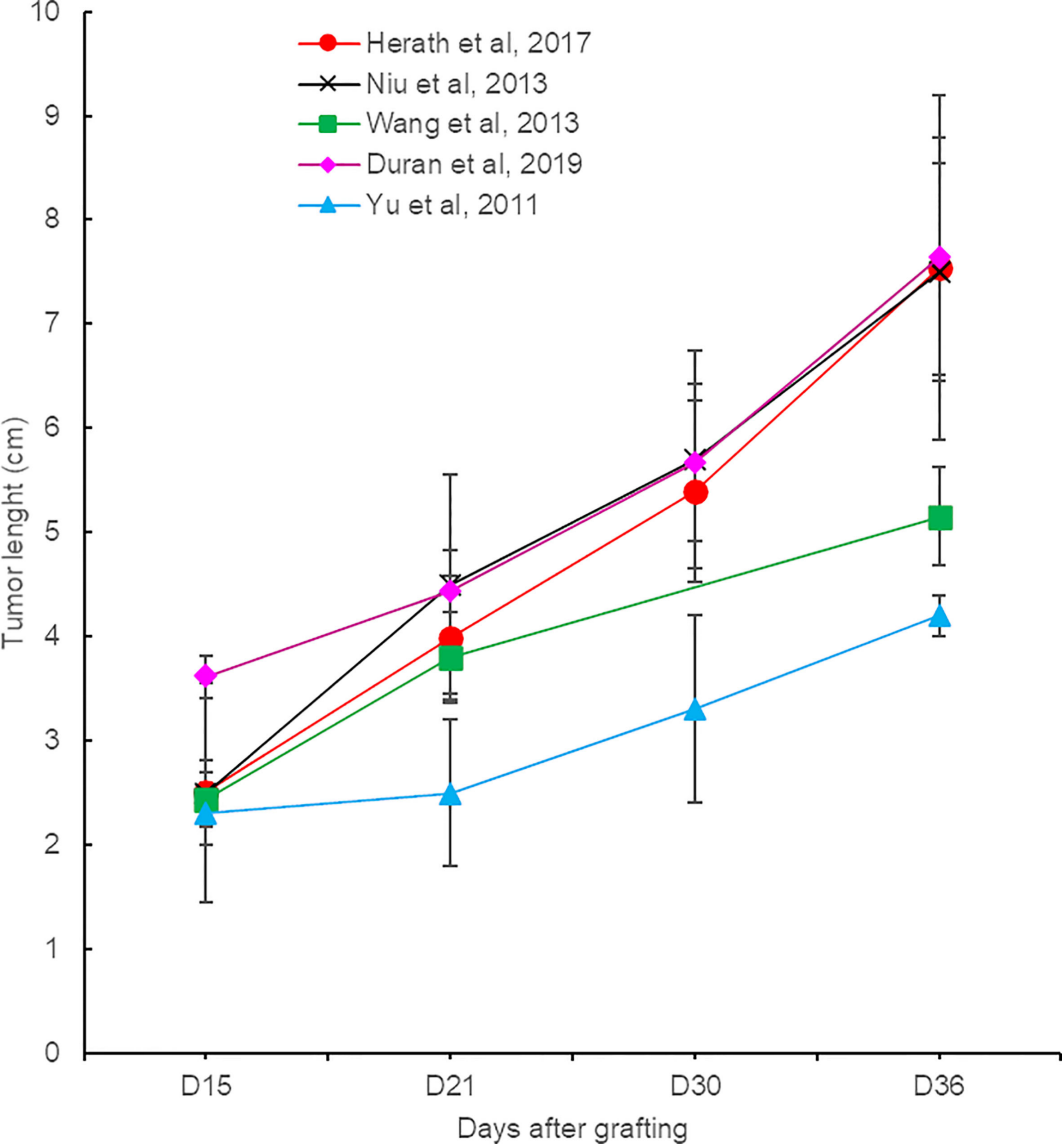
Evolution of VX2 liver tumor size from different reports.

Tumor growth is fast, with a doubling of their volume every week (**Figure 2**). Two weeks after inoculation which is usually the time when the experiment is performed, the tumor reaches approximately 2.0 cm in length (2.5–5 cc in volume) (19–21). A size of 3 cm, which is considered a threshold for surgery/ablation vs. TACE allocation in hepatocellular carcinoma (HCC) patients, is reached after approximately 15 days, depending on the modality of tumor induction. At 6 weeks after implantation, rabbits may present very large tumors measuring 7.5 cm in length and 115 cc in volume (7, 12, 17, 19, 22, 23).

### 1.2 Animal Follow-Up

Rabbits generally show no clinical signs of the disease for a period of 1 month after tumor inoculation. They have a normal behavior and appetite, and no clinical signs of pain are observed. Liver function is not impaired despite tumor burden. The biochemical parameters of the liver and kidney (liver enzymes alanine transaminase (ALT) and alkaline phosphatase (ALP), creatinine, and urea nitrogen) remain in the normal range for that period of observation (17, 22, 24). After 36 days, animals become less brisk with a selective appetite. They may show signs of abdominal pain, due to possible ascites, peritoneal metastases, and tumor-associated cytokines (17, 25) and trouble breathing because of pulmonary invasion. Neutrophils are highly increased at 36 days (>10,000/µl), and this neutrophilia is strongly associated with the development of lung metastasis (22, 26). Other biology parameters appear unaffected.

### 1.3 Metastatic Spread

**Figure 3:**
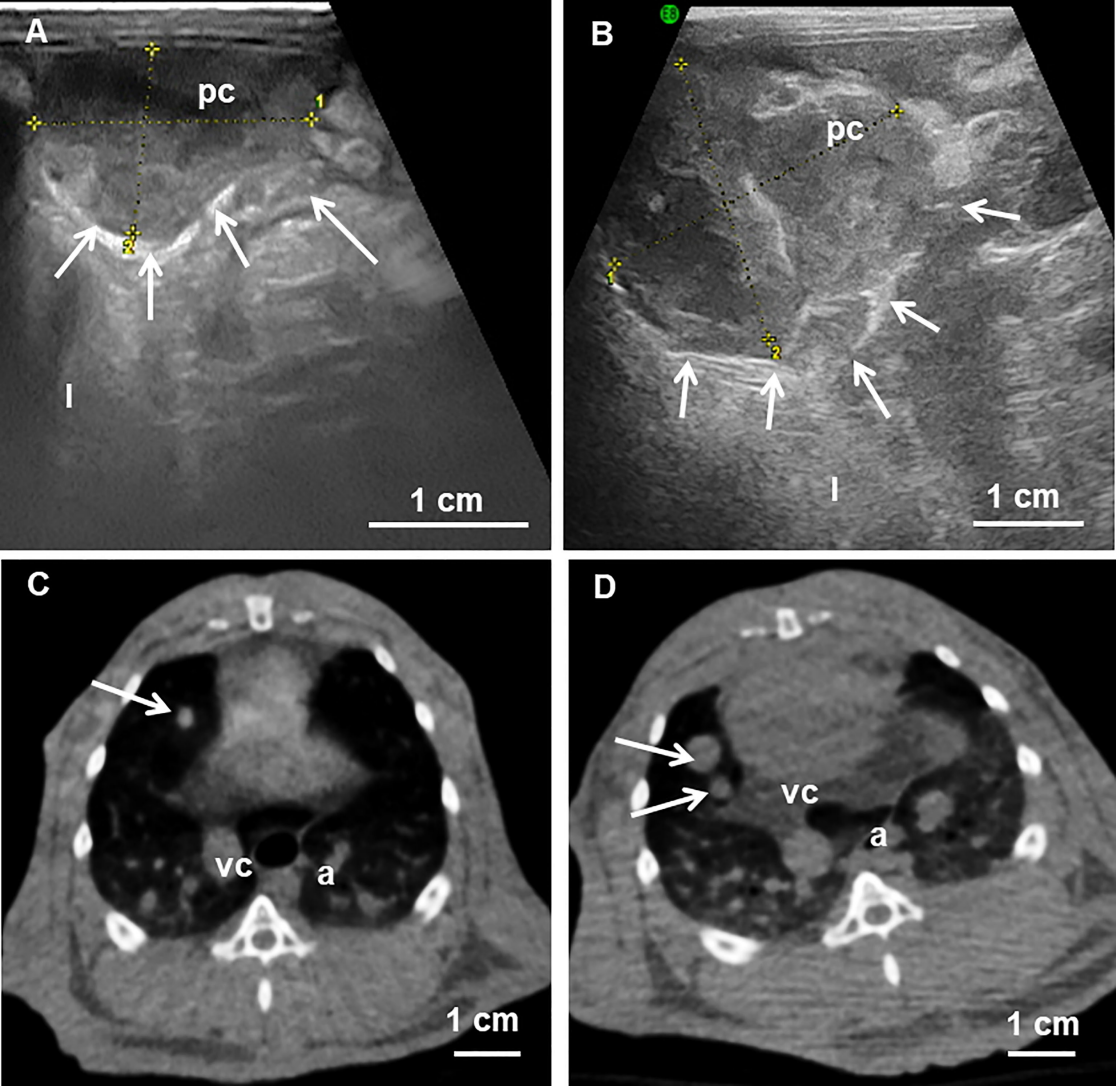
**(A, B)** Abdominal ultrasound examination 30 and 36 days after tumor inoculation showing abdominal peritoneal metastases and peritoneum surrounding the carcinosis (arrows). The metastases increased in size and became more invasive over time. **(C, D)** Cone beam CT at the thoracic level shows small pulmonary nodules (arrows) at 18 days that have increased in size one week later. pc, peritoneal carcinosis; l, liver; vc, vena cava; a, aorta. From (22).

Although rarely described in literature, abdominal and pulmonary metastases should be expected (**Figures 3A–D**). Early discovery is likely due to tumor seeding at the time of implantation and may be detected in 16% for lungs and 8% for peritoneum 2 weeks after liver inoculation. Extra-hepatic nodules may also develop due to tumor spread at later time points. After 6 weeks, 89% to 100% of animals not receiving any treatment will develop metastases (17). They are the main factor limiting the duration of follow-up of the animals.

### 1.4 Survival

Survival is the most important outcome of an anticancer therapy. This is rarely an endpoint reported in VX2 preclinical trials because the tumor growth and metastases are life-threatening for the control untreated animals. The reported average survival time for the untreated animals is 45 days after tumor inoculation (12, 21, 24, 25, 27). After this period, the general state of the animal becomes impaired by the tumor burden and/or by the complications associated with pulmonary spread.

VX2 animals undergoing anticancer therapy are fine at 45 days without any clinical sign of the disease and can survive much longer than 45 days. The lifespan of VX2-bearing animals was extended up to 300 days in a study comparing different routes of administration of an anticancer agent mixture (28).

## 2 Vascularization

As in patients, the liver parenchyma in rabbit receives dual vascularization with 75%–80% of the blood supply coming from the portal system and 20%–25% from the hepatic artery while tumors are vascularized mainly by the hepatic artery (13). The liver arterial anatomy is also very close between rabbits and human. In patients, the hepatic artery commonly arises from the celiac trunk but may originate from the superior mesenteric artery in 3% of cases (29). In rabbit, angiography shows that the common hepatic artery also emerges from the celiac trunk in the majority of cases (98%) and in a few cases from the cranial mesenteric artery (2%) (30). In the two species, the common hepatic artery gives some digestive branches before ending into the liver as a proper hepatic artery (31). Inside the liver, the proper hepatic artery divides into two main branches, right and left hepatic arteries distributed to the right and left lobes, respectively. In rabbits, the left hepatic artery measures approximately 1 mm (0.6–1.5 mm) and divides into one or two branches that feed the tumor (30). The main tumor feeding artery measures 0.7 mm (0.5–0.9 mm), and the second feeding artery, arising from the left hepatic or from the principal feeder artery, has a diameter around 0.5 mm (0.3–0.8 mm). For comparison, the mean diameter of the main artery irrigating HCC nodules in human with a diameter of 7–63 mm was measured between 0.1 and 1.8 mm (32).

The diameter of intratumoral vessels can be deduced from embolization trials that have evaluated the distribution of embolic particles in histology or imaging (33–35). In VX2, beads with a caliber below 100 µm are mostly located inside the tumor nodule, microspheres with a size between 100 and 300 µm are evenly distributed inside and outside the tumor, and particles larger than 300 µm may not penetrate inside the tumor bed. Interestingly, two studies demonstrated a similar distribution of beads in HCC explants (36, 37), suggesting that the size of intratumoral vessels may be in the same range for patient hepatomas and VX2 tumors.

Tumor vascularization depends on the tumor size. Below a diameter of 2.5 cm, the vessels are homogenously distributed into the tumor core and capsule (38). When the tumor size exceeds 3 cm, the feeding artery becomes larger and the vessels are dense at the tumor edges but the core of the tumor becomes poorly vascularized. In patients, this aspect is frequently observed for the liver metastases from gastrointestinal adenomas (39).

## 3 Imaging

### 3.1 Morphology

**Figure 4:**
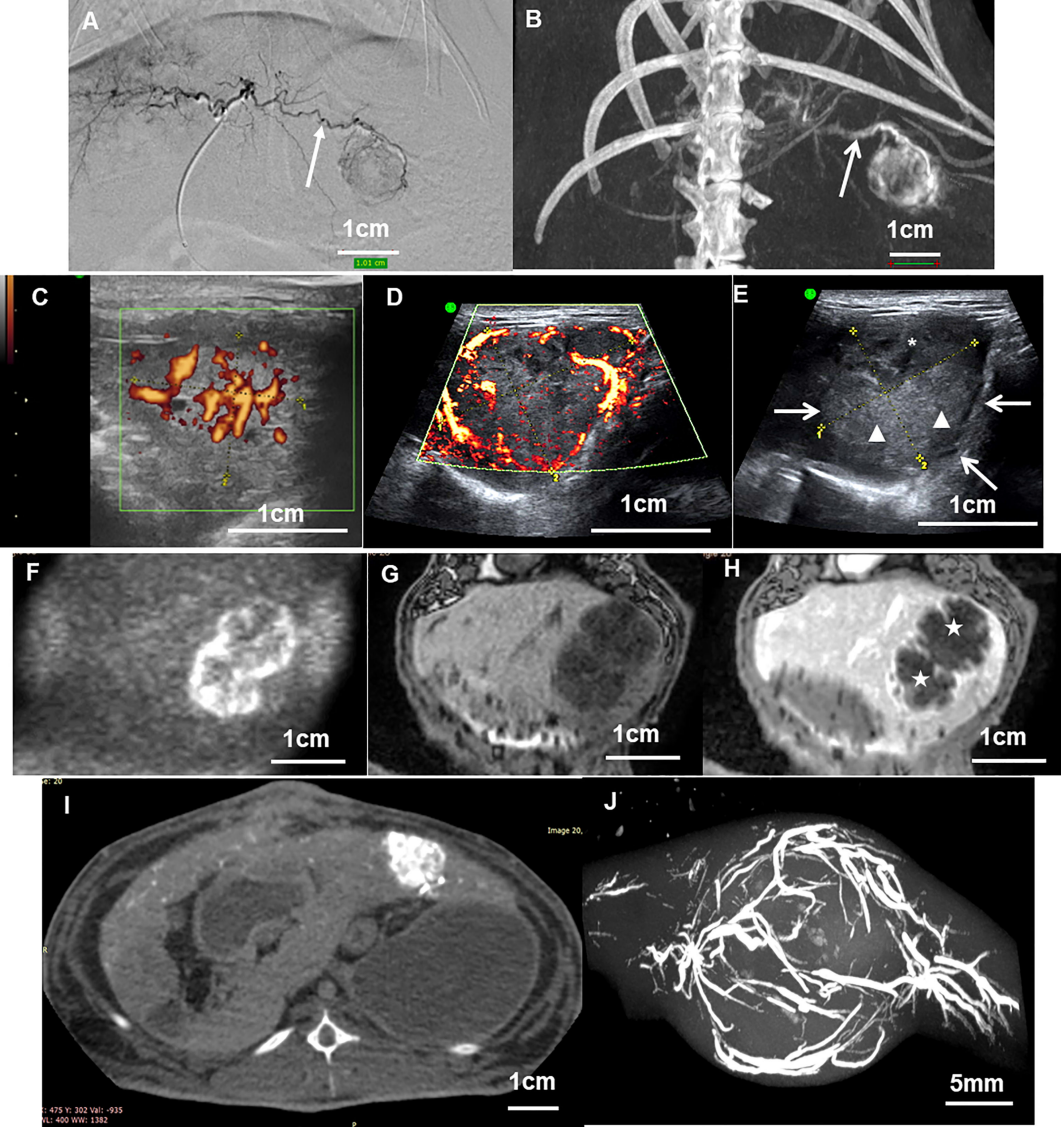
**(A)** Angiography and **(B)** cone beam CT acquisitions of a VX2 tumor after 13 days of tumor development showing the main tumor feeding artery (arrow). **(C)** Ultrasound image in Power Doppler mode of VX2 tumor at 13 days showing vessels inside the tumor core. **(D)** The same examination of the same tumor at 21 days showing vessels at the periphery of the nodule. **(E)** The same tumor in B mode gray scale showing the tumor as a heterogenous mass with hyperechogenic (arrow heads) and hypoechogenic (stars) areas that correspond to viable and necrotic areas respectively, and hypoechogenic aspect of the tumor boundaries (arrows). **(F–H)** Coronal MRI view of a 21-day tumor with diffusion weight imaging **(F)**, T1-weighted before **(G)** and after **(H)** intravenous Dotarem contrast injection. The vessels are enhanced by gadolinium injection at the tumor boundaries while the necrotic core of the tumor remains unenhanced (stars). **(I)** Axial slice of a cone beam CT abdominal acquisition of a rabbit showing enhanced VX2 liver tumor after intra-arterial contrast injection. **(J)** Maximum intensity projection of a micro-CT acquisition of a VX2 tumor injected intra-arterially with radiopaque beads. Beads are seen with high attenuation in the vessels inside and around the nodule. All figure parts are from (16, 22).

The aspect of VX2 tumors under different imaging modalities has been described in many different publications (**Figures 4A–J**). For morphology assessment, ultrasound is usually preferred due to the easy access to the equipment, affordability, and the fact that the examination can be executed on an awake rabbit. Tumors are identified as a heterogenous mass with hyperechogenic and hypoechogenic areas that correspond to viable and necrotic areas, respectively, and the hypoechogenic aspect of the tumor boundaries. The liver around appears as a uniform, sponge-like texture of low-level gray. The demarcation between the tumor and normal liver parenchyma is sometimes difficult and needs an experienced operator or the injection of contrast (40).

In computed tomography (CT), VX2 liver tumors have a low-density appearance which resembles the normal liver and cannot be clearly distinguished from surrounding parenchyma without contrast injection (41).

In magnetic resonance imaging (MRI), the VX2 morphology is better depicted by T2-weighted imaging, as a mass with well-defined margins and areas of high and low signals corresponding to viable and necrotic portions, respectively (42). The liver around appears in the lower signal compared to the tumor.

### 3.2 Viability

Recent papers have tried to develop functional imaging tools mainly to evaluate the viability of the tumor or its perfusion after treatment.

Viability may be investigated by different MRI protocols with or without exogenous markers. The apparent diffusion coefficient (ADC) in diffusion-weighted imaging (DWI) is increased in areas of tumor necrosis compared to viable tumor regions at the tumor margin (38, 42). The concentration of choline and lipids as determined by hydrogen-1 proton MR spectroscopy may also reflect the percentage of tumor necrosis (43). The use of contrast agents specific for components of the extracellular matrix allowed a finer visualization of baseline tumor morphology as well as fibrotic remodeling of the periablation zone after radiofrequency treatment (44). VX2 tumors have an increased glucose metabolism and reduced oxidative metabolism, resulting in acidosis of the tumor microenvironment that promotes the tumor growth, metastasis, and resistance to therapy. By generating an extracellular pH map by specific MRI procedures, the proliferating portions of the tumor or areas that do not respond to treatment can be visualized (45). Apoptosis could also be evaluated *in vivo* in VX2 tumors by positron emission tomography (PET) with the use of ^18^F-labeled Annexin V targeting the phosphatidylserine exposed on damaged cellular membranes (46).

### 3.3 Vascularity

The vascularization and perfusion of VX2 liver tumors can be evaluated by many imaging modalities. Power and color Doppler sonography provides a basic and easy-to-perform macrovascular assessment in a semiquantitative approach and has been shown to anticipate tumor response after chemoembolization (17). A quantitative assessment of the tumor angiogenesis was established by quantitative three-dimensional (3D) dynamic contrast-enhanced ultrasound (DCE-US) (40).

Tumor vascularity can also be assessed by contrast-enhanced CT using a perfusion protocol. Quantitative parameters can be evaluated such as arterial flow, portal flow, and perfusion index (47). The VX2 tumors display a marked enhancement in the arterial phase, while the necrotic core of the tumor and the surrounding normal liver parenchyma appear unenhanced. In the portal phase, the tumors show low opacity and the liver around is strongly enhanced, giving the best visualization of the tumor (41). Spectral CT can be used for the quantitative evaluation of tumor angiogenesis (27).

Dynamic contrast-enhanced MRI (DCE-MRI) measures several parameters to quantitatively assess tissue vessel density, integrity, and permeability (48, 49) and further allows the visualization of hypoxic areas of the tumors (50). Transcatheter intra-arterial perfusion (TRIP) MRI was developed as an arterial analog to DCE-MRI to help quantify hepatic arterial perfusion in tumors (51).

Finally, the microvascularity and neo-angiogenesis of VX2 tumors could be nicely pictured by MRI using contrast media that target molecules involved in endothelial cell sprouting (52).

## 4 Histopathology

### 4.1 Histology

**Figure 5:**
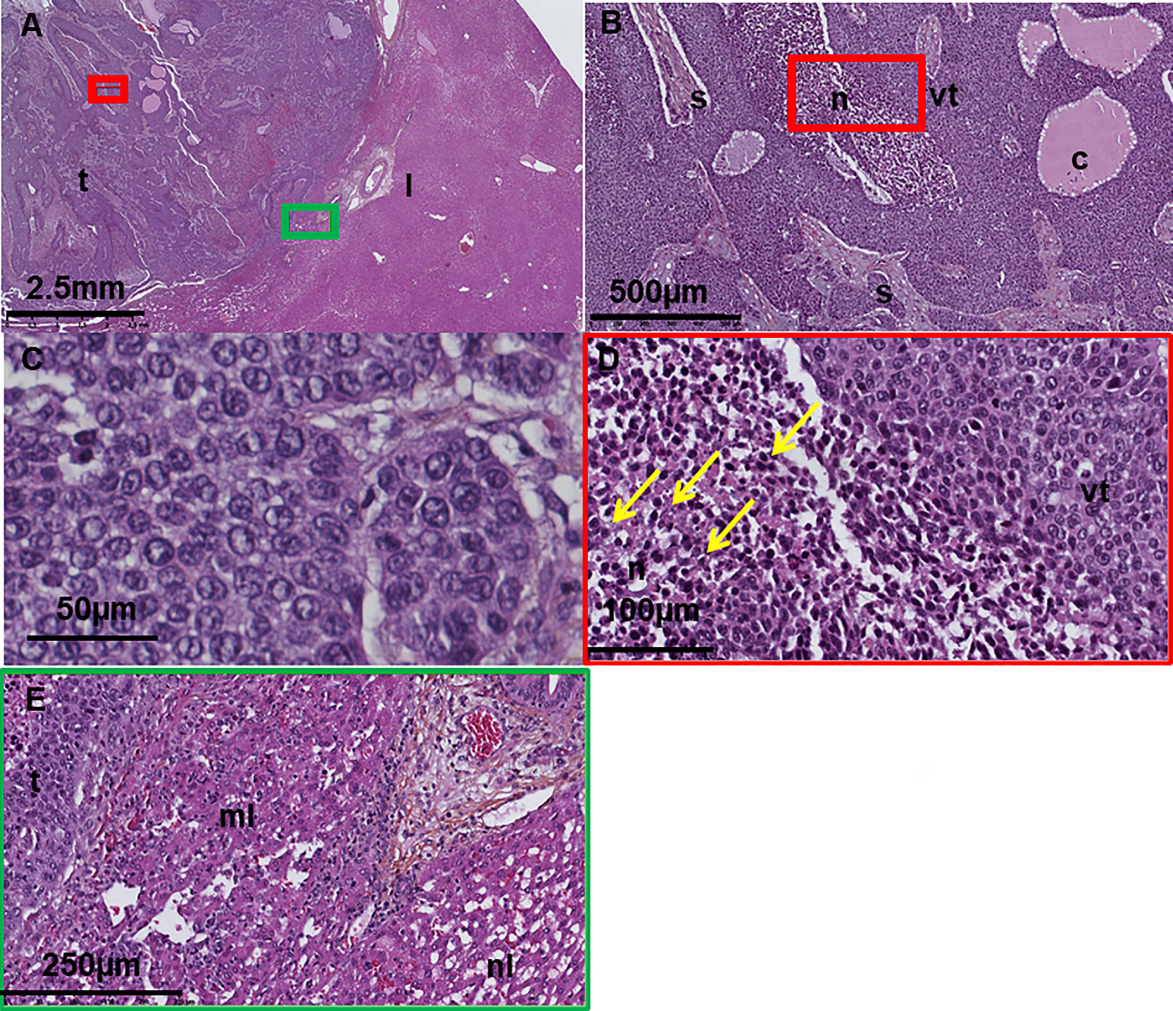
**(A–E)** Digitized histology section of a VX2 tumor in the liver at different magnification showing **(A)** normal liver and tumor, **(B)** areas of viable tumor, necrosis, cystic cavities and fibrous stroma, **(C)** large undifferentiated tumor cells with high nucleo-cytoplasmic ratio, eosinophilic/pale cytoplasm, and ill-defined borders. **(D)** Apoptotic cell debris (arrows) in the necrotic areas. **(E)** Compressed and normal liver in the vicinity of the tumor t, tumor; l, liver; n, necrosis; vt, viable tumor; c, cyst. From (22, 55).

The histology of VX2 is well known (1, 53, 54). Tumors are generally well delineated and composed of sheets and lobules of large undifferentiated cells with a high nucleo-cytoplasmic ratio (**Figures 5A–E**). Their nuclei are large, round, and with moderate anisocaryosis, coarse chromatin, and inconspicuous nucleolus. Their cytoplasms are eosinophilic or pale with ill-defined borders resulting in a pseudo-syncytial aspect. Mitosis and apoptotic bodies are numerous, the later tending to concentrate in the center of the lobules. The largest tumor sheets or lobules are generally centered by necrotic areas containing apoptotic-cell debris. Cystic cavities of various sizes, containing a proteinaceous eosinophilic fluid and some apoptotic/necrotic debris, are often present. The tumors are surrounded and penetrated by various, generally low, amounts of fibrous stroma, containing some blood vessels and few inflammatory cells, mostly macrophages and lymphocytes (56). The surrounding liver is normal or may show compression-distorted hepatic plates in the vicinity of the tumor.

Control non-treated animals will show a fraction of dead tumor due to spontaneous necrosis that may reach 30%–40% of the tumor surface the third week after tumor development (34, 38). The percentage of necrotic tumor may increase with tumor size (38). This spontaneous necrosis is often considered a limitation of the model and requests the inclusion of a non-treated or sham group to discriminate the effects of the tested therapy itself.

### 4.2 Immunohistochemistry

**Figure 6:**
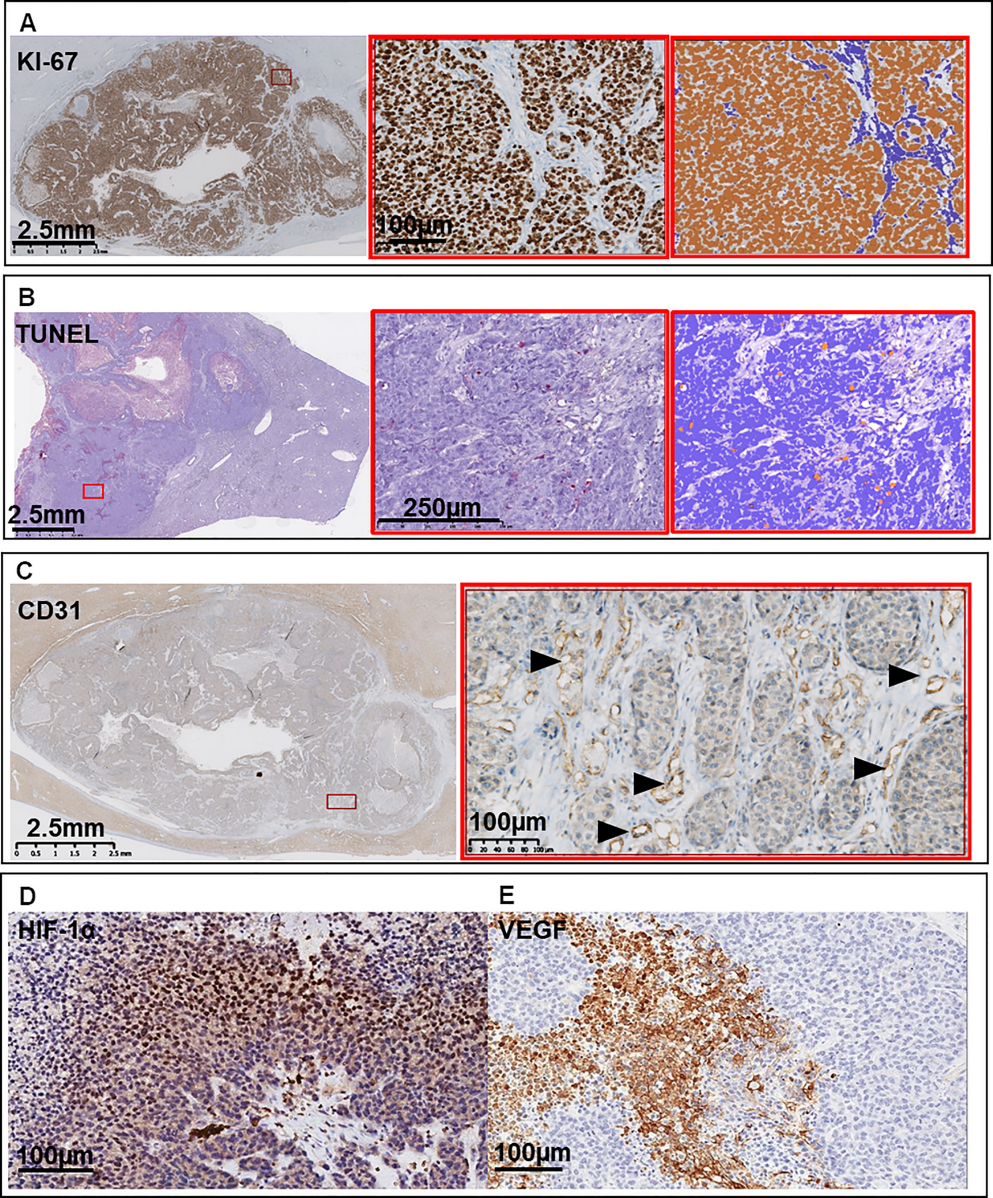
Representative immunohistochemistry images of **(A)** Ki-67 labeling for proliferating cells at low magnification, high magnification, and automatic counting with ImmunoRatio plug-in for ImageJ. **(B)** TUNEL for apoptotic cells at low magnification, high magnification, and automatic counting with ImmunoRatio plug-in for ImageJ. **(C)** CD31 for endothelial cells (arrow heads), **(D)** hypoxia-inducible factor HIF-1α, and **(E)** vascular endothelial growth factor VEGF. From (7, 22).

In immunohistochemistry, VX2 tumors are positive for cytokeratin AE1/AE3 and high molecular weight cytokeratin (HCK) labeling and negative for low molecular weight cytokeratins and CK18 (57, 58), as reported for the majority of squamous cell carcinomas (**Figure 6**). They also show strong, diffuse staining for the proliferation markers Ki-67 and PCNA all over the tumor surface and positive scattered labeling for vimentin, which are characteristic of aggressive cancers. The VX2 tumors are also positive for ALDH1 and CD44, two markers of cancer stem cells (59).

Regarding the tumor microenvironment, a positive immunohistochemical signal could be detected for different members of the matrix metallopeptidase family (MMP2, MMP3, MMP9, TIMP2, TIMP3) which are key players in the tumor invasion neovascularization processes, especially where tumors actively proliferated (60, 61). In non-treated tumors, epithelial cancer cells may represent 75% of viable cells, while CD11b+ macrophages and CD8+ T cells compose the majority of the non-tumoral cells (58, 62). To our knowledge, subtyping of other immune cells has not been explored.

Immunohistochemistry with CD31 and CD34 markers for endothelial cells has also shed light on the microvascularity of the liver tumors. Vessels are mostly present in the capsule and outer part of the tumor while the microvascular density is lower in the center of the tumor (40, 63). As a consequence, overexpression of hypoxia-inducible factor 1 alpha (HIF1α) is primarily found in tumor cells in proximity to the tumor core (45). Histopathologic markers indicative of glycolysis (GLUT-1) and chronic acidosis (LAMP-2) were found to be upregulated in untreated VX2 tumors (45).

Contrary to HCC, the basal levels of heat-shock protein HSP70 in VX2 tumors are low (64). The expression of other diagnostic markers for primary [glypican 3 (GPC3), glutamine synthetase (GS), arginase 1, hepatocyte paraffin 1 antigen (Hep Par-1)] and secondary [caudal-type homeobox 2 (CDX2), special AT-rich sequence-binding protein 2 (SATB2)] liver tumors has not been explored by immunohistochemistry.

## 5 Tumor Microenvironment

**Figure 7:**
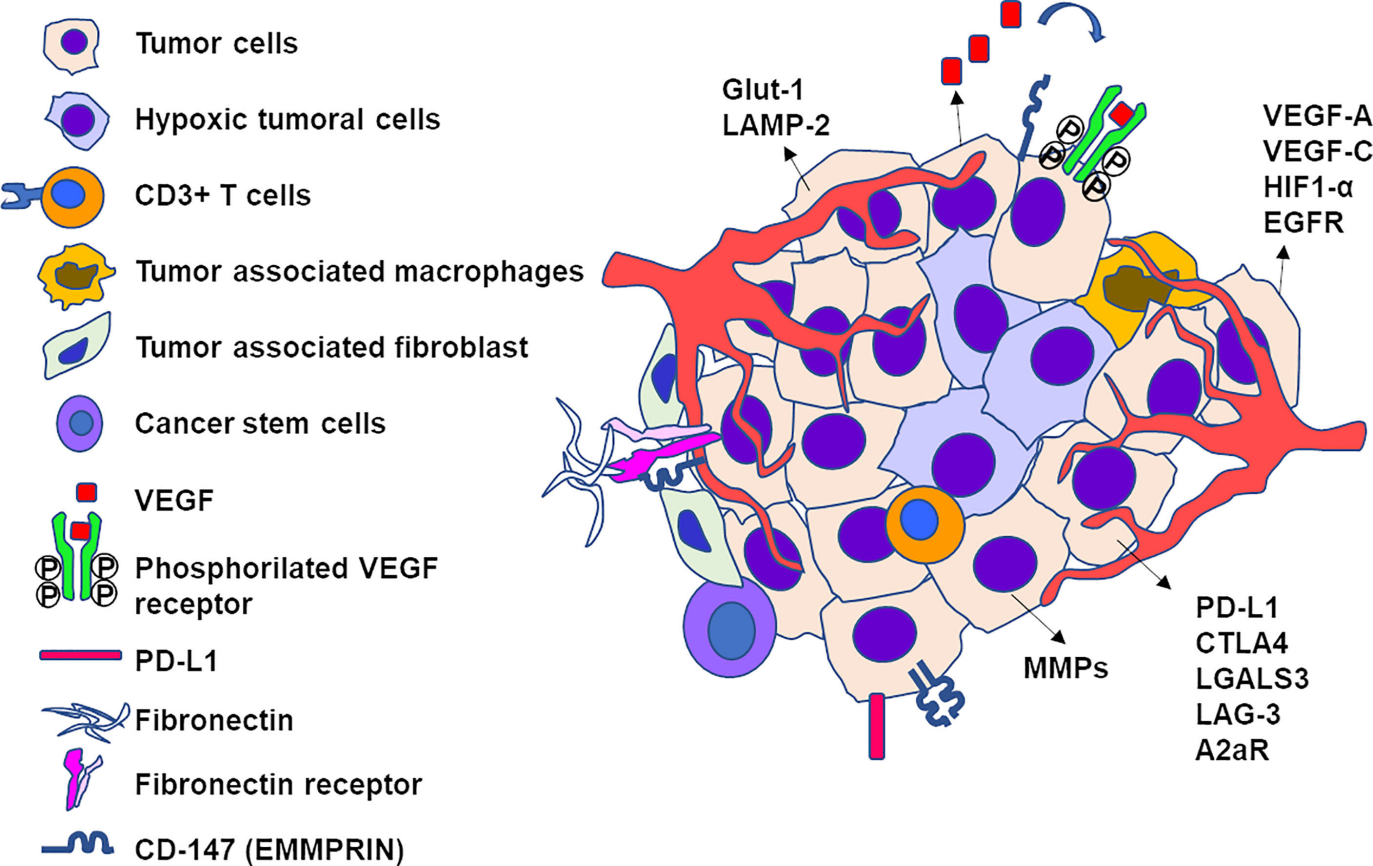
Schematic representation of elements of tumor microenvironment of an untreated VX2 tumor. Viable tumor cells are found at the periphery of the nodule while hypoxic cells locate in the center. The following non-tumor cells have been found in tumor microenvironment: CD3+ T cells, tumor-associated macrophages, tumor-associated fibroblasts, and cancer stem cells. The mediators and receptors overexpressed by the tumor include markers of angiogenesis (VEGF-A, VEGF-C, EGFR, HIF-1α), immunologic checkpoints (PDL-1, CTLA-4, Gal-3, LAG-3, A2aR), extracellular matrix (fibronectin receptor, MMPs, CD147).

The last two decades reported a switch in concept of cancer therapy, from therapies focusing on the tumor itself to therapies centered on its microenvironment. The tumor microenvironment (TME) designates cancer cells, stromal cells, blood vessels, nerve fibers, extracellular matrix, and associated acellular components located at the center, at the margin, or within the vicinity of the tumor lesion. It can be classified into different specialized microenvironments (65), namely, immune microenvironment, hypoxic and acidic niche, mechanical microenvironment, innervated niche, and metabolism microenvironment. The VX2 tumor model is scarcely used for basic research on TME. However, several early and recent therapeutic studies have revealed some of the signaling pathways and molecules involved in the TME of this tumor (**Figure 7**). We will focus on the three first types of microenvironment which have been more explored in VX2.

### 5.1 Immune Microenvironment

Livers bearing primary or secondary tumors are characterized by multiple immune regulatory changes that act in favor of (liver intrinsic immunotolerance, immunosuppression of chronic inflammation) or against (antitumor response) disease progression. The goal of cancer immunotherapy is to shift that balance toward immunity against the tumor, using different non-exclusive strategies: blocking the suppressor lymphocytes that maintain the tolerance of the immune system for the tumors (checkpoint inhibitors), making some hidden tumor antigens visible to immune cells, or repopulating the tumors with cytotoxic lymphocytes and turn “cold” cancers into “hot” ones. Several of these approaches have been tested in VX2 tumors, as presented below.

#### 5.1.1 Blockade of Immune Checkpoint Inhibitors

The role of several immune checkpoints including programmed cell death 1/programmed death-ligand 1 (PD-1/PD-L1), cytotoxic T-lymphocyte-associated protein 4 (CTLA-4), lymphocyte-activation gene 3 (LAG-3), adenosine A2a receptor (A2aR), or T-cell immunoglobulin and mucin domain-3 (TIM-3) has been highlighted in the immune tolerance for HCC (66). All these markers are highly upregulated on CD4+ and CD8+ cells infiltrating the HCC and are predictive of poorer disease outcome and postoperative recurrence. The first success of immunotherapy has been recently achieved in HCC with the approval of the anti-PD-L1 atezolizumab in combination with anti-VEGF bevacizumab as first-line treatment for advanced diseases.

In VX2, the expression of PD-L1, CTLA-4, LAG-3, and A2aR is significantly upregulated inside the tumors compared to the host liver tissue (67), suggesting that VX2 could potentially respond to immune checkpoint inhibitors. To our knowledge, no investigation on immune checkpoint inhibitors has been reported in VX2. One study did investigate the effects of combined blocking of PD-1 and LAG-3 in a rabbit model of ocular herpes. They showed an increased number of functional tissue-resident antivirus-specific CD8+ T cells, associated with a protection against further infection (68).

#### 5.1.2 Unmasking Tumoral Antigens

**Figure 8:**
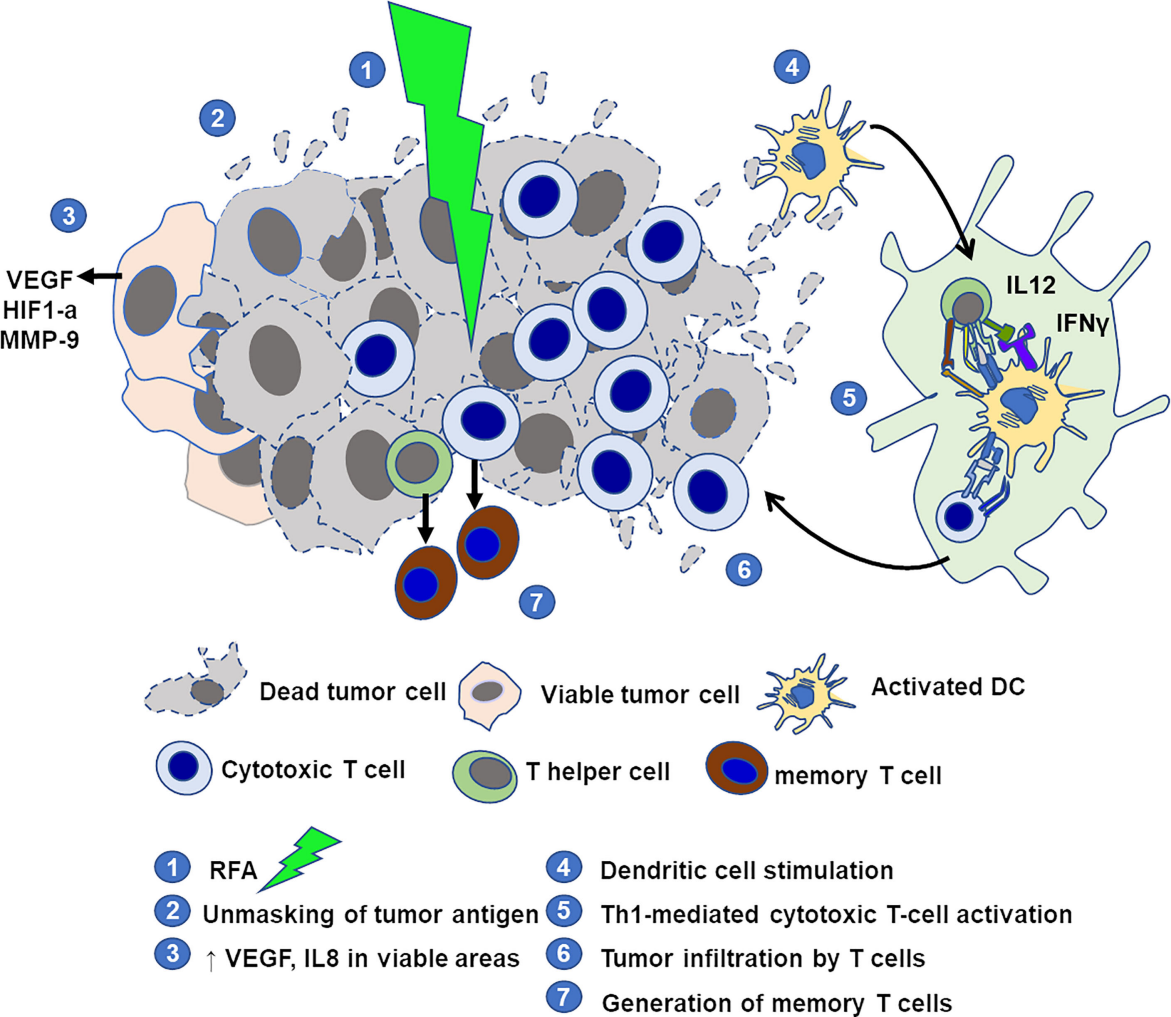
Schematic representation of elements of immune microenvironment in VX2 tumor treated by RFA with immunostimulation. RFA destroys the tumor and produces tumor-specific antigens. Areas at the periphery of the tumor may remain viable and show increased expression of pro-angiogenic factors. Adjuvant stimulation of dendritic cells with CpG promotes a tumor-specific Th1 and cytotoxic T-cell reaction, increases the number of lymphocytes infiltrating the tumor, and allows the generation of memory T cells.

Despite its immunotolerance, VX2 is an immunogenic tumor. In 1978, Shah et al. demonstrated several key facts on the immunocompetence after locoregional therapy using the rabbit VX2 liver model (69). First, the local hyperthermia treatment of the tumor elicits an immune response against tumor extracts. This response is both cellular and humoral. It disappears over time in animals not showing a complete response and is maintained in respondent animals which do not show any tumor development upon rechallenging with *de novo* inoculation of VX2 cells. Since then, the immunomodulatory and abscopal effects of the locoregional treatments have been confirmed with different therapies. Radiofrequency ablation (RFA) induced prolonged survival of the treated animals versus controls. This survival advantage was correlated with the presence of an antitumoral T-cell response after RFA that made some cryptic tumoral antigens accessible to antigen-presenting cells (APC) (70). Locally, CD3+ T cells were observed infiltrating the tumor nodule. Peripheral blood cells from these animals further showed an increase in activation when exposed to tumor lysates as well as specific increased cytotoxicity when co-incubated with tumor cells. This immune stimulation is most likely directed against tumor antigens and not against transplantation alloantigens from donor animals, since non-treated VX2 animals do not show any immune reaction to tumor cells or tumor lysates. Adding a stimulation of dendritic cells by Toll-like receptor 9 (TLR9) ligands to RFA further potentiated antitumor T-cell response: tumor spread to other organs was prevented, survival was significantly prolonged compared to single treatments, and animals receiving a secondary tumor cell injection did not develop any tumor (25), suggesting a memory immune response against the tumor (**Figure 8**).

In another study, peritoneum-ozonized oxygen insufflation was applied to rabbits with VX2 auricular tumors. Regressing tumors showed an increased number of intratumoral CD3+ T cells and overexpression of genes coding for antigen presentation, T-cell activation, and inflammatory mediators. Most interestingly, the injection of peripheral blood leukocytes from responder rabbits to newly implanted animals resulted in tumor regression, showing that this oncolytic immune response may be adoptively transferred (71).

#### 5.1.3 Stimulation the Homing of T Cells

Another immunomodulatory approach has been tested in VX2 tumors, that is, the reinforcing of the impotent immune system of the host. The delivery of interferon gamma inside the tumor led to a significant increase in natural killer cell infiltration *via* C–X–C motif chemokine ligand 10 (CXCL10)-mediated migration (72). Peritumoral injection of interleukin 2 (IL2), a potent stimulator of the helper and cytotoxic T cells, caused complete rejection for 33% of animals of both the treated auricular tumors and the non-treated contralateral tumors, and acquired immune rejection upon rechallenging. Similar results were obtained after intra-arterial injection of IL2 to rabbits with liver metastases of colorectal cancer (73). Several studies have also achieved better tumor response and systemic antitumor immunity when a locoregional therapy was associated with an immunostimulant treatment (74–76).

### 5.2 Hypoxia Niche and Angiogenesis

Hypoxia is an important hallmark of cancer and its microenvironment. It is involved in tumor angiogenesis, progression, stemness, intercellular communication, or resistance to treatment. It is described as a common feature of solid tumors including hepatocellular carcinoma and globally associated with poor prognosis. Two mediators have been particularly emphasized, vascular endothelial growth factor (VEGF) and hypoxia-inducible factor 1 alpha (HIF-1α). Overexpression of VEGF has been observed in HCC, and the concentration of circulating VEGF correlates with advanced HCC tumor stage, with the highest level observed in patients with metastases (77). The protein level of HIF-1α is significantly elevated in HCC samples and associated with worse prognosis, but the expression of mRNA shows variations. Intravascular therapies may induce an elevation of VEGF serum levels which is a negative prognostic factor for treatment outcome (77).

The basal expression of VEGF and HIF-1α in VX2 tumors has been evaluated at transcriptomic and protein levels. VX2 tumors constitutively express VEGF-A, the main type of growth factor and target for bevacizumab involved in tumor angiogenesis and growth (56, 78), as well as VEGF-C, responsible for the morphogenesis of lymphatic vessels and metastasis development (67, 79). The protein may be found in the plasma at very low concentrations (<10 pg/ml). VEGF receptor 2 is also present in active phosphorylated form in non-treated tumors (55), showing the activation of angiogenesis *via* p38 MAPK, PI3K/AKT, and/or PLC-MEK/ERK pathways. HIF-1α is overexpressed in proximity to the necrotic tumor core which is poorly vascularized and deprived of oxygen supply (45, 80). Similarly to patients, chemoembolization of VX2 liver tumors induces ischemia, an increase in HIF-1α, and hypoxia which trigger the synthesis of VEGF (81, 82). These effects actually depend on the aggressiveness of treatment. The expression of HIF-1α may decrease immediately after cTACE and remain undetectable several weeks after TACE along with the onset and increasing extent of tumor necrosis. On the contrary, undertreated tumor portions after incomplete treatment have characteristic viable tumor features with the overexpression of HIF-1α and other markers of tumor progression (45). Perfusion maps of embolized VX2 tumors proved that embolization until angiographic stasis eliminated perfusion in only 56% of microvessels (83), further suggesting that partial response of the tumor and pro-angiogenic effects of embolization may be due to incomplete devascularization of the tumor.

Several therapies that block the induction of angiogenesis pathway have been tested in VX2, e.g., by combining chemoembolization with an oral tyrosine kinase inhibitor or by delivering an antiangiogenic drug rather than a cytotoxic drug through TACE procedure. TACE with sorafenib, sunitinib, apatinib, or vandetanib was successful at inhibiting the angiogenesis, tumor growth, and metastatic spread of VX2 tumors (22, 55, 84, 85). Interestingly, groups treated with the anti-angiogenic drug in these trials often showed partial efficacy, suggesting that the drugs are active in this preclinical model. A more recent therapeutic approach has proposed to take advantage of this adverse hypoxic effect of TACE and inject prodrugs which will be activated by the non-normoxic environment, with promising results in the VX2 model (7).

**Figure 9:**
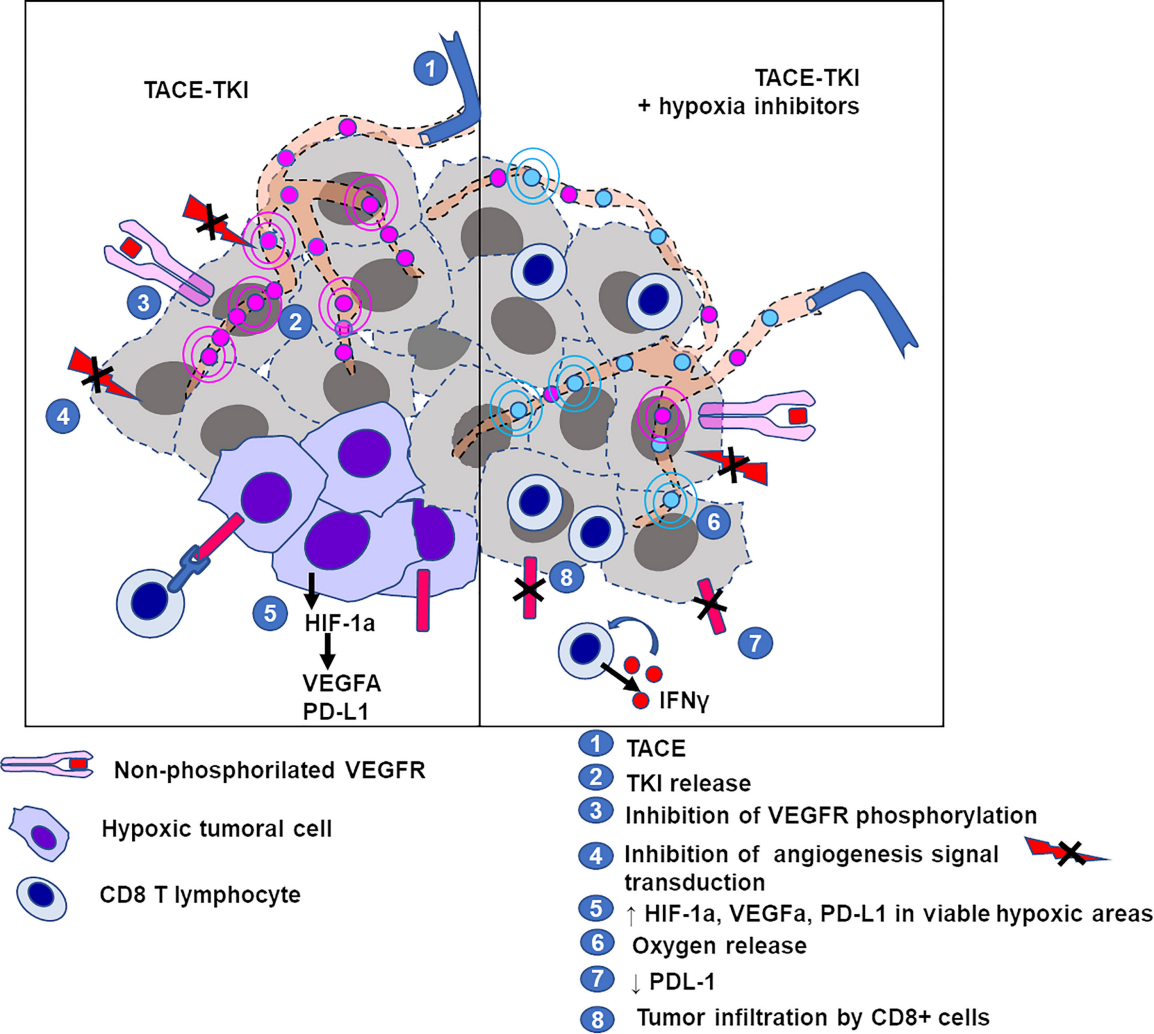
Schematic representation of elements of the tumor microenvironment in VX2 tumor treated by TACE with/without hypoxia inhibitor. Left panel: TACE with tyrosine kinase inhibitors (TKI) induces ischemia plus inhibition of angiogenesis *via* blockade of TK receptor phosphorylation, leading to tumor cell death. Some areas of the tumor may remain viable under hypoxic conditions due to embolization and show overexpression of HIF-1α, markers for tumor progression and immunosuppression. Right panel: counteracting the hypoxia (with oxygen release mediated by catalase) downregulates the expression of PD-L1, allowing the activation and infiltration of CD8+ cytotoxic T cells.

The hypoxic niche inside the tumor also has a negative impact on its immune microenvironment, by excluding effective immune cells from the acidic and poorly vascularized regions of the nodule (**Figure 9**). Normalization of tumor extracellular pH using bicarbonate or oxygen-generating catalase during TACE counteracted these immunosuppressive effects and triggered the homing of the cytotoxic lymphocytes, resulting in tumor regression (45, 86).

### 5.3 Mechanical Microenvironment

The tumor microenvironment also includes the factors of the extracellular matrix (ECM). Matrix metalloproteinases (MMPs), the principal ECM-degrading enzymes, are often overexpressed in cancer and are associated with a poor prognosis. The MMP receptor CD147 (EMMPRIN) is a glycoprotein initially known as a regulator of MMPs through cell–matrix and cell–cell interactions which have been identified as a potential target for cancer therapy (87). In HCC, the chimeric anti-CD147 humanized antibodies inhibited invasion and metastasis by modulation of cytoskeleton rearrangement *via* the FAK–PI3K–AKT signaling pathway (88).

As in HCC, MMP9 detection in VX2 liver tumors is associated with rapid progression of the tumor, especially after locoregional therapies such as TACE and RFA (89, 90). Targeting CD147 with the (131)I-labeled CD147 antibody prolonged survival and inhibited the tumor growth and metastasis spread in VX2 liver tumors (23).

Fibronectin, the main component of the ECM that is particularly abundant in tumors, ligates the integrin α5β1 on both tumor and other cells of ECM, especially vascular endothelial cells. This interaction induces tumor growth and invasion by activation of the Akt and MAPK pathways. Volociximab, a human/mouse chimeric antibody against integrin α5β1, inhibits endothelial cell proliferation and induced cell death. Despite a lower affinity of the antibody to the rabbit antigen, volociximab administered systemically to VX2-bearing animals still resulted in a significant decrease in tumor volume (91).

In the tumor microenvironment, cancer stem-like cells (CSC) are a subpopulation of cells with elevated tumor-initiating potential. They have been identified in both human liver neoplasms and the rabbit tumor (59). It was demonstrated that reducing the genomic instability in VX2 with the use of a protector (aminoethyl isothiourea) decreased tumor-initiating cells. When combined to chemotherapy, this strategy decreased lung metastases and prolonged survival compared to that with the cytotoxic agent alone (92).

Altogether, these reports support the production of tumor-specific antigens after conventional locoregional treatment of liver VX2 and show that immunity against tumor can be developed by adding different types of immunotherapy. Immune response is better in responder animals and may lead to recovery or even tumor rejection upon rechallenging of the animals.

Questions remain regarding the utility of the rabbit VX2 in immuno-oncology compared to other tumor models. Basic knowledge about the cancer immunity is still missing, e.g., immune and non-immune cell populations of the microenvironment are still poorly characterized. While some key mediators of the immune microenvironment have been identified, few molecules have been tested in the model. The availability of the antibodies used in targeted and IO treatment and the knowledge of their cross-reactivity with the rabbit antigens remain a major challenge. Finally, different pathways probably exist between human and rabbit tumors (carcinogenetic process, interaction with inflamed/healthy liver tissue), despite the similarities demonstrated here. A high-throughput analysis of genomic, transcriptomic, and proteomic data from the tumor could help in specifying their common and unique features.

## Conclusion

The rabbit VX2 carcinoma has been extensively used as a model of liver tumor to assess various treatment modalities that required animals larger than rodents. Its implantation techniques or growth characteristics have been accurately described, but information about its biology is scattered. The present review aimed at giving a faithful resource to guide teams working in interventional oncology and help them in the design of their future preclinical investigations.

With an appropriate surveillance of biology parameters and metastatic development, survival seems an acceptable endpoint for efficacy studies using the model. Many imaging protocols allow the morphology and functional characterization of the tumor with the same equipment as per clinical practice. With a particular interest in the tumor microenvironment, we showed that similar cells, mediators, mechanisms, and effects can be observed between human cancer and VX2. Of note, therapies aiming at immunizing the rabbit against the tumor are the only treatments that achieved a complete response and no recurrence.

In conclusion, VX2 carcinoma has a place alongside other experimental cancer models and seems to be the most relevant for trials combining locoregional treatments and therapies targeting the tumor microenvironment.

## Author Contributions

FP is the first author and the corresponding author. JN is the last author. J-PP, MW, SG, J-PS-M, TB, AD, RD, FD, OP, NM, and AL have contributed equally to this work. All authors contributed to the article and approved the submitted version.

## Funding

The authors declare that this study received funding from Archimmed SARL. The funder was not involved in the study design, collection, analysis, interpretation of data, the writing of this article or the decision to submit it for publication.

## Conflict of Interest

FP, SG, and JN are paid employees of Archimmed SARL. J-PP, MW, AL, and JN are shareholders of Archimmed.

The remaining authors declare that the research was conducted in the absence of any commercial or financial relationships that could be construed as a potential conflict of interest.

## Publisher’s Note

All claims expressed in this article are solely those of the authors and do not necessarily represent those of their affiliated organizations, or those of the publisher, the editors and the reviewers. Any product that may be evaluated in this article, or claim that may be made by its manufacturer, is not guaranteed or endorsed by the publisher.
